# Metabolic Signatures of Resistance to Mycobacterium Tuberculosis Infection: Insights from a Multi-Country Plasma Metabolomics Study

**DOI:** 10.64898/2026.09.29.26364337

**Published:** 2026-10-05

**Authors:** Chang Liu, Jeffrey M. Collins, Matheus Fernandes Gyorfy, Mariana Araújo Pereira, Vidya Mave, Senbagavalli Prakash, Kamakshi Prudhula Devalraju, Neil A. Martinson, Fay Willis, Marina C. Figueiredo, Marcelo Cordeiro-Santos, Artur Trancoso Lopo de Queiroz, Venkata Sanjeev Kumar Neela, Rajesh Karyakarte, Timothy R. Sterling, Jerrold J. Ellner, James C.M. Brust, Amita Gupta, Bruno B. Andrade, Yan V. Sun, Neel R. Gandhi

**Affiliations:** 1 Department of Epidemiology, Emory University Rollins School of Public Health, Atlanta, Georgia, USA; 2 Emory University School of Medicine, Atlanta, Georgia, USA; 3 Multinational Organization Network Sponsoring Translational and Epidemiological Research (MONSTER) Initiative, Salvador, Brazil; 4 Byramjee Jeejeebhoy Government Medical College - Johns Hopkins University Clinical Research Site, Pune, India; 5 Center for Infectious Diseases in India, Johns Hopkins India, Pune, India; 6 Jawaharlal Institute of Postgraduate Medical Education and Research, Puducherry, India; 7 Bhagwan Mahavir Medical Research Centre, Hyderabad, India; 8 Perinatal HIV Research Unit, University of the Witwatersrand, Johannesburg, South Africa; 9 Department of Medicine, Vanderbilt University Medical Center, Nashville, Tennessee, USA; 10 Universidade do Estado do Amazonas, Manaus, Brazil; 11 Universidade Nilton Lins, Manaus, Brazil; 12 Fundação de Medicina Tropical, Manaus, Brazil; 13 Laboratório de Pesquisa Clínica e Translacional, Instituto Gonçalo Moniz, Fundação Oswaldo Cruz, Salvador, Brazil; 14 Byramjee Jeejeebhoy Government Medical College and Sassoon General Hospitals, Pune, India; 15 Department of Medicine, Division of Infectious Diseases, Rutgers New Jersey Medical School, Rutgers Health, Newark, New Jersey, USA; 16 Division of General Internal Medicine, Albert Einstein College of Medicine, Bronx, NY, USA; 17 Johns Hopkins School of Medicine, Baltimore, Maryland, USA; 18 Johns Hopkins Bloomberg School of Public Health, Baltimore, Maryland, USA; 19 Atlanta VA Healthcare System, Decatur, Georgia, USA.

**Keywords:** Mycobacterium tuberculosis, tuberculosis resistance, metabolomics

## Abstract

**Background:**

Tuberculosis (TB) remains the leading infectious disease cause of mortality worldwide. A subset of individuals exposed to *Mycobacterium tuberculosis* (*Mtb*) remain TST/IGRA-negative despite sustained exposure, termed “TB resisters”. The biological mechanisms underlying this resistance remain poorly understood. We applied untargeted high-resolution metabolomics to determine whether systemic metabolic profiles differ between TB resisters and matched *Mtb*-infected close contacts.

**Methods:**

We compared untargeted high-resolution plasma metabolomics using liquid chromatography mass spectrometry between 269 TB resisters and 269 matched *Mtb*-infected close contacts, enrolled across Brazil, India, and South Africa. TB resisters were defined as highly exposed close contacts who slept in the same room or spent at least 5 hours indoors per day with an infectious pulmonary TB index patient, but remained TST/IGRA-negative. *Mtb*-infected close contacts were those who were TST/IGRA positive. Metabolome-wide association studies (MWAS) were conducted using paired t-tests.

**Results:**

There were 1,787 features with nominal differences between TB resisters and matched *Mtb*-infected contacts (p < 0.05). Pathway enrichment identified fructose and mannose metabolism and bile acid biosynthesis in the overall cohort, while lipid-related pathways were enriched in Brazil. Among metabolites with confirmed chemical identities, glyceric acid concentrations were higher in TB resisters by 0.53 μM, whereas hydroxyproline was lower by 0.90 μM, butyrobetaine was lower by 0.06 μM, and homogentisate was lower by 0.0036 μM at false discovery rate of 0.2.

**Conclusions:**

Plasma metabolomic profiles differed between TB resisters and matched *Mtb*-infected close contacts. These findings indicate that systemic metabolic differences are associated with resistance to *Mtb* infection.

## Introduction

Tuberculosis (TB) remains the leading infectious disease cause of death worldwide and continued transmission highlights the need for strategies that prevent *Mycobacterium tuberculosis* (*Mtb*) infection ^1^. A longstanding observation is that some close contacts of infectious TB patients remain uninfected, with persistently negative tuberculin skin test (TST) or interferon-γ release assays (IGRAs) despite sustained exposure. While it is unclear whether these “TB resisters” never become infected, clear bacilli early or become *Mtb* infected without an interferon gamma response ^2,3^, long-term follow-up data show that these individuals have a very low 10-year risk of developing TB disease or developing a positive TST or IGRA test ^4^. Identifying biologic mechanisms associated with this long-term protection may allow for the development of new prevention tools, including a new TB vaccine.

Metabolomic profiling captures the integrated biochemical consequences of immune activation and cellular energetics, providing a functional readout of immunometabolic pathways that may influence early *Mtb* clearance ^5,6^. Prior TB metabolomics studies have largely contrasted active TB with latent *Mtb* infection or more broadly defined “exposed/uninfected” comparators, and have repeatedly implicated disruptions in tryptophan-kynurenine catabolism, lipid remodeling and acylcarnitine metabolism, as well as amino acid metabolism ^7-9^. However, few studies have evaluated rigorously ascertained TB resisters, despite evidence that this phenotype reflects distinct host biology ^2,10,11^. To address this gap, we conducted a multi-country, matched resister-control study nested within close contact cohorts in India, Brazil, and South Africa. We applied untargeted high-resolution plasma metabolomics using the samples collected at enrollment to compare highly exposed contacts who remained persistently TST/IGRA-negative (“TB resisters”) with matched contacts with evidence of *Mtb* infection (TST/IGRA-positive). We hypothesized that TB resisters would exhibit distinct systemic metabolic profiles compared with *Mtb*-infected controls.

## Methods

### Study Population

Participants for this study were drawn from the parent TB genome-wide association study (GWAS) cohort, which enrolled 4,058 close contacts of pulmonary TB patients from nine prospective cohorts across 10 sites in Brazil, India, and South Africa, in collaboration with the RePORT consortia; 4,000 participants had sufficient data to determine resister/control status **Supplemental Table 1** ^12,13^. Detailed study methods of the parent TB study have been previously published ^13^. Briefly, close contacts of microbiologically confirmed (e.g., positive culture and/or Xpert) pulmonary TB index patients were eligible for enrollment if they: (i) resided in the same household for ≥1 month, or (ii) spent ≥4 hours per week with the index patient before the index patient’s diagnosis. Close contacts were excluded if they were HIV-coinfected or unable to provide informed consent/assent.

Characterization of TB resister and control phenotypes are detailed in **Supplemental Methods**. Briefly, we created eight phenotypic categories representing a hierarchy of resistance and infection, based on index patient infectiousness, exposure to the index patient, and IGRA and/or TST test results **Supplemental Figure 1**. We identified TB resisters among participants with high exposure (slept in the same room or ≥5 hours indoors) to a highly infectious index case (cavitary disease or 2+/3+ smear grade), who were persistently TST and/or IGRA negative were categorized as Resister A, B, or C categories, **Supplemental Figure 1**. Controls were selected from participants with a high likelihood of being *Mtb*-infected. These included contacts with positive TST and/or IGRA results or a history of TB disease, categorized as Infected A if both IGRA and TST were positive, and Infected B if either IGRA or TST was available and positive, **Supplemental Figure 1**.

In the current metabolomic study, we analyzed a subset of 538 participants from the parent TB GWAS study. Participants with a high likelihood of being resistant to *Mtb* infection (Resister A n = 35; Resister B n = 234; Resister C n = 0; total n=269) were matched 1:1 to participants with a high likelihood of being *Mtb*-infected (Infected A n = 77; Infected B n = 192; total n=269). For each Resister participant, a matched control was sought who shared the same index patient and who was similar in age. When matches with the same index patient were not possible, control participants with a similar age, same sex and within same country of enrollment were sought, **Supplemental Methods**.

All participants provided written informed consent/assent. Institutional review boards at Emory University and all participating sites approved the study, **Supplemental Methods**.

### Untargeted High-Resolution Metabolic Profiling

Baseline plasma samples were analyzed using previously established methodologies ^14-18^. The plasma specimens were collected, stored at −80°C, and thawed prior to analysis by liquid chromatography-mass spectrometry (LC-MS). To prepare the samples, aliquots were treated with acetonitrile containing an isotopic internal standard mix, then centrifuged at 4°C for 10 minutes to remove proteins. The resulting supernatant was kept in an Accela Open Autosampler at 4°C for subsequent analysis. Triplicate analyses were performed using liquid chromatography coupled to high-resolution Orbitrap mass spectrometry (Q Exactive, Thermo Fisher Scientific), acquired over an m/z range of 85-1100 with a 5-minute run time. Extracts were analyzed using HILIC with positive electrospray ionization and reverse-phase C18 chromatography with negative electrospray ionization. HILIC and reverse-phase C18 chromatography were used as complementary platforms to broaden metabolomic coverage, with HILIC enriching detection of polar metabolites and C18 improving detection of less polar and lipid-associated metabolites. HILIC separation was performed using a Waters XBridge BEH Amide XP HILIC column. C18 separation was performed using an endcapped Higgins Targa C18 column. The software apLCMS was utilized for metabolic feature extraction ^19^. A metabolic feature is defined by m/z (mass-to-charge ratio) and retention time. Only features showing a Pearson correlation coefficient greater than 0.7 across all triplicates were retained to ensure data quality. The intensities of metabolic features were summarized as median values of the non-zero readings across triplicates. Features with >50% missing values were excluded from downstream analyses. The remaining zero readings were considered values under detectable range and imputed as random values below the half of the minimum intensities across all samples. Intensities were then log2-transformed (log2(intensity+1)), mean-centered, and scaled by standard deviation.

Annotation of features was performed using xMSannotator ^20^ matching to the Human Metabolome Database ^21^. xMSannotator assigns confidence scores (0-3) by integrating multiple orthogonal pieces of evidence, including expected isotope/adduct relationships, retention-time clustering and intensity correlation patterns across samples, and network/pathway context, and then applying chemical plausibility filters ^20^. Annotations with a confidence score of 3 were classified as highly confident, and those with a score of 2 as moderately confident. Only annotations with a score of 2 or higher were used. In addition, a subset of metabolites were further confirmed based on established standards ^15,22^. Metabolite identification and confirmation were carried out using an in-house metabolite library, which was validated through comparisons of retention time and MS/MS data with authentic standards, as well as by matching MS/MS fragments with online spectral databases. The library was then used, allowing a tolerance of ± 5 ppm for m/z and ± 30 seconds for retention time, relative to standardized plasma, for accurate metabolite annotation. The intensities of confirmed metabolites were converted to concentrations in uM using a previously described protocol ^15^.

### Statistical Analysis

Subject characteristics were described as median (interquartile range) for continuous variables and frequency (percentage) for categorical variables. These characteristics are described for the overall cohort and stratified by study site, including Brazil, India, and South Africa.

For the untargeted metabolome-wide association study (MWAS), paired t-tests were conducted to compare the metabolite intensities between the paired TB resisters and matched controls for each metabolic feature. The paired t-test was chosen to account for the within-pair nature of the comparison. Statistical significance was assessed using a threshold of p < 0.05. To account for multiple testing, the Benjamini-Hochberg false discovery rate (FDR) ^23^ method was used with FDR corrected q value < 0.2 defined as statistical significance ^24,25^. For metabolites with the highest confidence annotations and concentrations, a separate analysis was performed on the concentrations using paired t-tests. Similarly, multiple testing correction was applied using the FDR approach. All analyses were performed in the overall cohort, and stratified by study site. Analyses were performed using R version 4.4.0 (R Foundation for Statistical Computing, Vienna, Austria).

Additionally, pathway enrichment analysis was performed following the untargeted MWAS using Mummichog (version 2.1.1) ^26^. Metabolic features that were different between TB resisters and non-resisters at a p value < 0.05 were chosen as target features and all features in the MWAS were utilized as the feature reference pool. Pathways with a FDR corrected q value < 0.2 were considered statistically significant.

## Results

The study included 269 matched pairs of TB resisters and *Mtb*-infected controls, including 155 pairs from Brazil, 103 from India, and 11 from South Africa, Table 1. By design, all resisters were QFT-negative and had high-intensity exposure to highly infectious index cases (cavities or AFB smear ≥2+/3+), with shared room/bed or ≥5 hours of indoor exposure. Across sites, males comprised 40% of resisters and 42% of controls; median age was 27-28 years overall (32-34 years in Brazil; youngest in South Africa at 13 years), and median BMI was 23.7 kg/m^2^ in resisters vs 23.1 kg/m^2^ in controls, highest in Brazil (24.3-24.6 kg/m^2^) and lowest in South Africa (17.2 kg/m^2^ in resisters; 14.5 kg/m^2^ in controls).

A total of 12,177 and 10,996 metabolic features, respectively for HILIC positive and C18 negative modes, passed quality control and entered the untargeted MWAS comparing resisters with controls. The overall cohort showed 911 features in HILIC and 876 in C18 significant at p < 0.05, with only 2 HILIC features significant at FDR<0.2. In the study site stratified MWAS, no features passed the FDR threshold, except for one in the South Africa cohort from the HILIC positive mode, **Supplementary Table 2, Supplementary Figures 2-5**. Among the metabolic features with p < 0.05, 307 in the overall cohort, 270 in Brazil, 260 in India, and 191 in South Africa had annotations and are presented in **Supplementary Tables 3-6**, respectively.

The pathway enrichment analysis of the untargeted MWAS identified 2 and 13 pathways in the analysis of overall cohort and Brazil, respectively. Fructose and mannose metabolism and bile acid biosynthesis were enriched in the overall cohort. Key lipid-related pathways, such as fatty acid activation, linoleate metabolism, omega-6 and omega-3 fatty acid metabolism, di-unsaturated fatty acid beta-oxidation were enriched in the Brazil cohort, Table 2, Figure 1.

A total of 127 metabolites were confirmed, and their quantified concentrations were analyzed. In the overall cohort, glyceric acid concentrations were higher in TB resisters than in controls (4.04 vs. 3.51 μM; mean difference, 0.53 μM; 95% CI, 0.18-0.88; P = 0.0032; FDR = 0.1997), whereas hydroxyproline (6.44 vs. 7.34 μM; mean difference, −0.90 μM; 95% CI, −1.51 to −0.29; P = 0.0041; FDR = 0.1997) and butyrobetaine (0.76 vs. 0.82 μM; mean difference, −0.06 μM; 95% CI, −0.10 to −0.02; P = 0.0047; FDR = 0.1997) were lower in TB resisters, Table 3. Notably, the direction of association for glyceric acid and butyrobetaine was generally consistent across Brazil and India, with no evidence of between-country heterogeneity (Pheterogeneity = 0.5667 and 0.5400, respectively). In contrast, hydroxyproline showed evidence of between-country heterogeneity (P heterogeneity = 0.0094), with a substantially larger inverse association in South Africa, although this estimate was based on only 11 pairs, Table 3. Country-specific analyses showed that homogentisate was lower in TB resisters than in controls in India (0.0189 vs. 0.0225 μM; mean difference, −0.0036 μM; 95% CI, −0.0057 to −0.0014; P = 0.0015; FDR = 0.1853), the only country-specific association meeting an FDR <0.20 threshold, Table 4.

## Discussion

This study investigated the metabolic differences between TB resisters and matched *Mtb*-infected controls using untargeted high-resolution metabolic profiling of plasma samples and additionally investigated confirmed metabolites using a targeted design. Pathway enrichment suggested involvement of carbohydrate metabolism, bile acid biosynthesis, and lipid-related pathways, particularly in the Brazil cohort. In the targeted study of metabolite concentrations, glyceric acid, hydroxyproline, butyrobetaine and homogentisate were significantly different between TB resisters and *Mtb*-infected controls. Together, these findings suggest that resistance to sustained *Mtb* infection may be associated with differences in systemic metabolic states related to energy metabolism, amino acid turnover, lipid handling, and oxidative or inflammatory processes.

Fructose and mannose metabolism intersects with central carbon metabolism and glycolytic flux, processes that are dynamically remodeled during macrophage responses to *Mtb* infection. Prior plasma metabolomics studies of pulmonary TB have also implicated fructose and mannose metabolism, glycolysis/gluconeogenesis, pyruvate metabolism, and bile acid biosynthesis as altered pathways in TB patients ^27^. The mannose-related signal is also biologically plausible in the context of *Mtb*-host interactions, as mannose-capped mycobacterial cell-wall components can engage macrophage mannose receptors and influence *Mtb* uptake and phagosome-lysosome trafficking ^28,29^. Similarly, bile acids are increasingly recognized as immunometabolic signaling molecules that regulate innate immune and inflammatory responses through bile acid-activated receptors ^30,31^. Thus, the enrichment of fructose/mannose and bile acid pathways may reflect systemic metabolic programs linked to macrophage activation, microbial sensing, or inflammatory regulation after high-intensity *Mtb* exposure. However, because these are pathway-level findings from cross-sectional plasma metabolomics, they should be interpreted as hypothesis-generating rather than evidence of causal mechanisms.

Our findings also highlight the critical role of lipid metabolism in shaping host immune responses to *Mtb*. This reflects differences in systemic lipid handling after high-intensity exposure, which may shape the metabolic milieu available to immune cells and influence antimicrobial programs. Intracellular *Mtb* can preferentially utilize host-derived fatty acids and cholesterol and can acquire a survival advantage in lipid-rich macrophage environments ^32-34^. In this context, enrichment of fatty-acid pathways could indicate a host state with altered lipid mobilization/turnover or lipid-mediator production that is associated with resistance. Notably, linoleate and omega-3/omega-6 pathways are upstream of bioactive lipid mediators that can modulate inflammation and macrophage fate decisions in TB, offering a mechanistic framework for how lipid signatures might relate to resistance ^35^. This interpretation is also consistent with prior work showing distinct monocyte transcriptional programs in highly exposed individuals who resist TST/IGRA conversion ^10,36^. Because our measurements are cross-sectional in plasma, we cannot determine whether these lipid-associated differences precede exposure or reflect very early host responses to exposure to *Mtb*; nevertheless, they nominate lipid metabolism as a key candidate axis associated with the TB resister phenotype that warrants longitudinal and cell-specific follow-up.

We also examined individual metabolites with high-confidence identifications, and several specific metabolites emerged as significantly different between resisters and *Mtb*-infected controls. One of the most notable was glyceric acid, which was elevated in resisters. Glyceric acid is a sugar acid produced from the oxidation of glycerol or intermediate steps of glycolysis ^37^, which are pathways that generate and use cellular fuel ^38^. Although glyceric acid itself has not been widely reported in TB, its higher levels in resisters may reflect the possibility that resisters differ in how rapidly they engage energy metabolism that supports innate immune activation. Hydroxyproline was significantly lower in resisters compared to *Mtb*-infected controls. Hydroxyproline is a non-standard amino acid released largely from the breakdown of collagen. It serves as a classic marker of collagen turnover and tissue remodeling ^39^. The lower plasma hydroxyproline in resisters implies less collagen degradation and tissue matrix remodeling in these individuals. Active TB disease is known to elevate hydroxyproline levels. A recent study found significantly higher hydroxyproline in the serum and even bronchoalveolar fluid of TB patients, correlating with inflammatory markers ^40^. This finding reinforces the notion that resisters did not develop an infection that triggered tissue reorganization. Butyrobetaine was lower in TB resisters, pointing to differences in carnitine-linked lipid metabolism. Butyrobetaine is the immediate precursor of L-carnitine, which supports mitochondrial transport and oxidation of long-chain fatty acids ^41^. Because butyrobetaine can also arise from microbial metabolism of dietary carnitine and feed into the TMA/TMAO pathway, this finding suggests potential host-microbiome influences on energy metabolism that warrant follow-up with longitudinal sampling and related metabolites ^42^. Homogentisate is formed from tyrosine metabolism, and previous study identified a link between host tyrosine metabolism and susceptibility to TB through genetic regulation of the *FAH* gene, which encodes the enzyme responsible for the final step of tyrosine catabolism.^43^ These results highlight tyrosine metabolism as a potential candidate pathway for future studies on TB and underscore the importance of integrating host genetics and metabolomics to uncover novel mechanisms of TB susceptibility.

The cross-sectional design limits causal inference, and metabolite differences may reflect consequences rather than causes of infection status. The modest sample size, particularly in South Africa, and inter-site variability may introduce heterogeneity. Further, plasma metabolomics may not fully capture lung-specific metabolic processes critical to early *Mtb* clearance, highlighting the need for longitudinal and tissue-focused follow-up studies. The major strength of the study is the use of a rigorously characterized, multi-ancestry cohort of TB resisters and matched *Mtb*-infected controls, enabling robust assessment of resistance to *Mtb* infection. The application of untargeted high-resolution metabolomics provided a comprehensive and unbiased view of systemic metabolic states, with rigorous quality control and pathway-level validation enhancing reliability.

## Conclusion

TB resisters showed distinct plasma metabolic patterns involving energy metabolism, bile acid biosynthesis, lipid-related pathways, and selected metabolites, including glyceric acid, hydroxyproline, butyrobetaine, and homogentisate. These findings support systemic metabolism as a candidate axis associated with resistance to sustained *Mtb* infection. Longitudinal and cell-specific studies are needed to validate these findings and determine whether these metabolic differences represent biomarkers or mechanisms of protection.

## Figures and Tables

**Figure 1.**
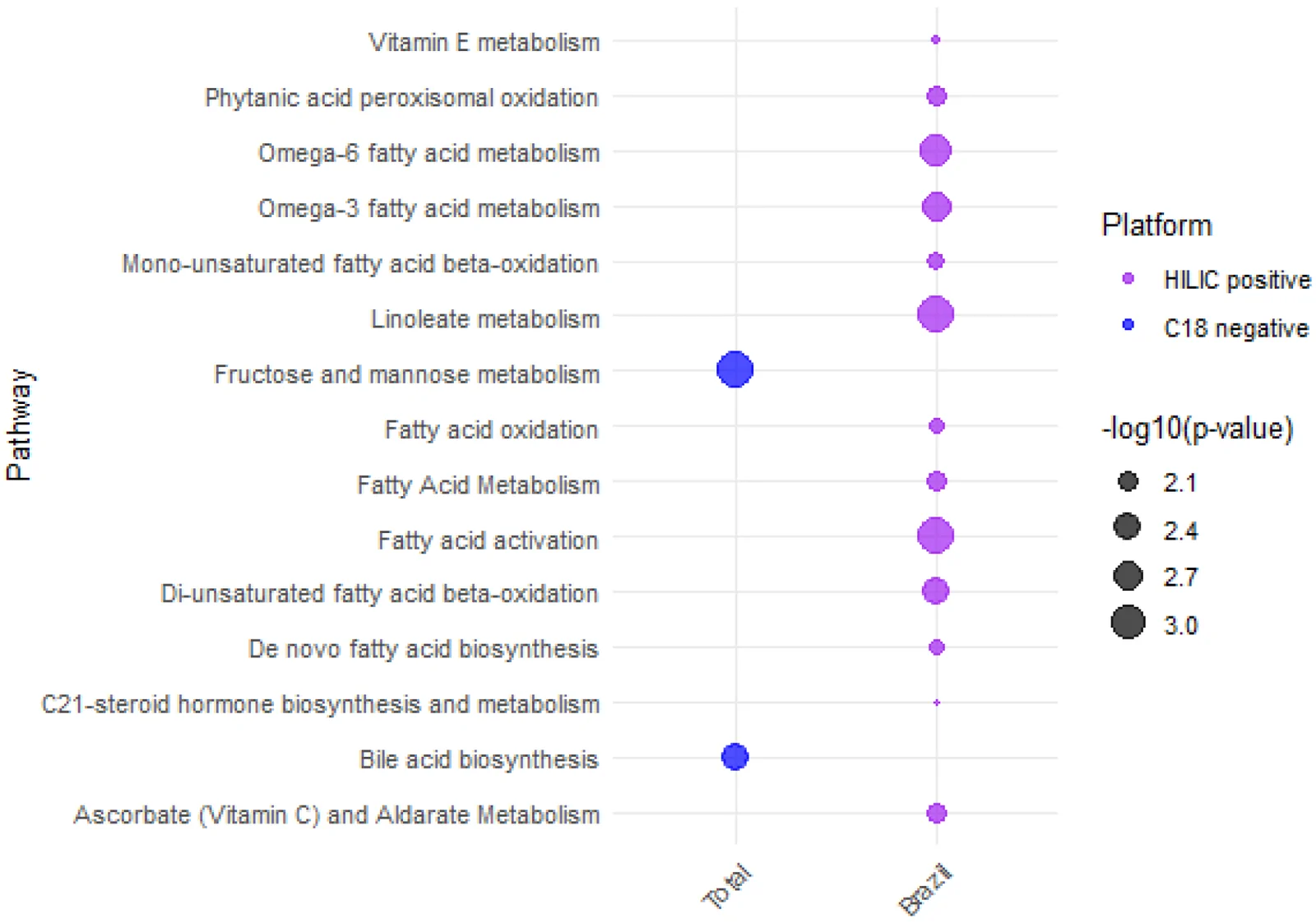
Pathway Enrichment Results of the Metabolome-wide Association Study Comparing TB Resisters and Controls.

**Table 1.** Characteristics of the Study Cohort.

Variable	Overall 269 Pairs		Brazil 155 Pairs		India 103 Pairs		South Africa 11 Pairs	
	Resisters N = 269	Controls N = 269	Resisters N = 155	Controls N = 155	Resisters N = 103	Controls N = 103	Resisters N = 11	Controls N = 11
**Age, years**	27 (13, 43)	28 (13, 42)	34 (15, 46)	32 (15, 46)	23 (12, 37)	25 (12, 38)	13 (12, 19)	13 (12, 19)
**Sex (%)**								
Female	161 (60)	155 (58)	92 (59)	94 (61)	60 (58)	53 (52)	9 (82)	8 (73)
Male	108 (40)	114 (42)	63 (41)	61 (39)	43 (42)	50 (49)	2 (18)	3 (27)
**BMI, kg/m^2^**	23.7 (18.7, 28.2)	23.1 (18.4, 27.9)	24.6 (20.6, 29.3)	24.3 (19.9, 28.7)	21.7 (16.8, 26.5)	20.3 (16.5, 25.2)	17.2 (14.6, 19.5)	14.5 (13.4, 19.8)
**QuantiFERON-TB Gold Test (%)**								
Negative	269 (100)	0 (0)	155 (100)	0 (0.0)	103 (100)	0 (0)	11 (100)	0 (0)
Positive	0 (0)	269 (100)	0 (0)	155 (100)	0 (0)	103 (100)	0 (0)	11 (100)
**Resistance & Infection Hierarchy Category**								
Resister A	35 (13)	0 (0)	0 (0)	0 (0)	35 (34)	0 (0)	0 (0)	0 (0)
Resister B	234 (87)	0 (0)	155 (100)	0 (0)	68 (66)	0 (0)	11 (100)	0 (0)
Resister C	0 (0)	0 (0)	0 (0)	0 (0)	0 (0)	0 (0)	0 (0)	0 (0)
Infected A	0 (0)	77 (29)	0 (0)	9 (6)	0 (0)	63 (61)	0 (0)	5 (45)
Infected B	0 (0)	192 (71)	0 (0)	146 (94)	0 (0)	40 (39)	0 (0)	6 (55)
**Index case infectiousness**								
High: Cavities present or AFB smear 2+/3+	269 (100)	201 (75)	155 (100)	114 (74)	103 (100)	85 (83)	11 (100)	2 (18)
Medium: AFB smear scanty/1+, OR GeneXpert grade high or very high (no cavities)	0 (0)	44 (16)	0 (0)	31 (20)	0 (0)	13 (13)	0 (0)	0 (0)
Low: AFB smear negative OR GeneXpert grade low or very low (no cavities)	0 (0)	16 (6)	0 (0)	10 (7)	0 (0)	5 (5)	0 (0)	1 (9)
Not available	0 (0)	8 (3)	0 (0)	0 (0.0)	0 (0)	0 (0.0)	0 (0)	8 (73)
**Exposure to index patient**								
Slept in same bed	53 (20)	38 (14)	35 (23)	17 (11)	18 (18)	20 (19)	0 (0.0)	1 (9)
Slept in same room	66 (25)	61 (23)	15 (10)	16 (10)	49 (48)	43 (42)	2 (18)	2 (18)
Spent ≥5 hours indoors together	150 (56)	83 (31)	105 (68)	55 (36)	36 (35)	21 (20)	9 (82)	7 (64)
None of the above	0 (0)	18 (7)	0 (0)	8 (5)	0 (0)	10 (10)	0 (0)	0 (0)
Not available	0 (0)	69 (26)	0 (0)	59 (38)	0 (0)	9 (9)	0 (0)	1 (9)

Median (interquartile range) shown for continuous variables. Count (percentage) shown for categorical variables.

AFB, acid-fast bacillus; BMI, body mass index; GeneXpert, Xpert MTB/RIF molecular test for Mycobacterium tuberculosis detection; TB, tuberculosis.

**Table 2.** Pathway Enrichment Results of the Untargeted Metabolome-wide Association Study Comparing TB Resisters and Controls. Metabolic Features with P Value < 0.05 Entered Pathway Analysis. Pathways with P Value < 0.05 and Overlapping Size ≥ 3 are Shown.

Cohort	Platform	Pathway	Number of Significant Metabolites	Number of Metabolites in Pathway	P	FDR
All Cohorts (269 pairs)	C18 negative	Fructose and mannose metabolism	8	12	0.0007	0.0800
	C18 negative	Bile acid biosynthesis	15	45	0.0032	0.1900
Brazil (155 pairs)	HILIC positive	Fatty acid activation	14	35	0.0006	0.0450
	HILIC positive	Linoleate metabolism	14	36	0.0008	0.0450
	HILIC positive	Omega-6 fatty acid metabolism	8	16	0.0017	0.0667
	HILIC positive	Omega-3 fatty acid metabolism	8	17	0.0025	0.0740
	HILIC positive	Di-unsaturated fatty acid beta-oxidation	5	8	0.0031	0.0740
	HILIC positive	Ascorbate (Vitamin C) and Aldarate Metabolism	9	26	0.0073	0.1244
	HILIC positive	Fatty Acid Metabolism	7	18	0.0085	0.1244
	HILIC positive	Phytanic acid peroxisomal oxidation	6	14	0.0085	0.1244
	HILIC positive	Mono-unsaturated fatty acid beta-oxidation	4	7	0.0094	0.1244
	HILIC positive	Fatty acid oxidation	5	11	0.0115	0.1291
	HILIC positive	De novo fatty acid biosynthesis	10	33	0.0119	0.1291
	HILIC positive	Vitamin E metabolism	6	16	0.0151	0.1400
	HILIC positive	C21-steroid hormone biosynthesis and metabolism	8	25	0.0153	0.1400

**Table 3.** Top Associations between Targeted Metabolites with Confirmed Annotations and TB Resistance in the Overall Cohort (269 Pairs). Quantified Concentrations in uM were Analyzed. Metabolites with P Value < 0.05 or False Discovery Rate < 0.2 in the Overall Cohort Analysis are Shown. Country-Specific Associations and Between-Country Heterogeneity for Targeted Metabolites Identified in the Overall Cohort are Shown.

Platform	m/z	time	Name	Adduct	All Cohorts (269 pairs)					Brazil (155 pairs)					India (103 pairs)					South Africa (11 pairs)					Heterogeneity Test P
					Resister Mean (SD)	Control Mean (SD)	Difference Resister vs. Control (95% CI)	P	FDR	Resister Mean (SD)	Control Mean (SD)	Difference Resister vs. Control (95% CI)	P	FDR	Resister Mean (SD)	Control Mean (SD)	Difference Resister vs. Control (95% CI)	P	FDR	Resister Mean (SD)	Control Mean (SD)	Difference Resister vs. Control (95% CI)	P	FDR	
C18 negative	105.0193	20.2	glyceric acid	M−H	4.04 (2.51)	3.51 (2.26)	0.53 (0.18, 0.88)	0.0032	**0.1997**	4.4 (2.5)	3.73 (2.43)	0.66 (0.15, 1.18)	0.0119	0.5046	3.59 (2.46)	3.19 (2.01)	0.4 (−0.06, 0.86)	0.0904	0.5669	3.31 (2.43)	3.47 (1.85)	−0.17 (−1.92, 1.59)	0.8358	0.9576	0.5667
HILIC positive	132.0656	70.7	hydroxyproline	M+H	6.44 (4.78)	7.34 (5.75)	−0.9 (−1.51, −0.29)	0.0041	**0.1997**	6.61 (4.21)	7.32 (4.31)	−0.71 (−1.58, 0.17)	0.1129	0.6034	5.77 (5.29)	6.36 (6.49)	−0.59 (−1.37, 0.19)	0.1349	0.6277	10.3 (5.64)	16.8 (7.88)	−6.51 (−10.23, −2.78)	0.0030	0.3812	**0.0094**
HILIC positive	146.1176	45.6	butyrobetaine	M+H	0.76 (0.41)	0.82 (0.42)	−0.06 (−0.1, −0.02)	0.0047	**0.1997**	0.77 (0.34)	0.84 (0.4)	−0.07 (−0.12, −0.02)	0.0056	0.5046	0.7 (0.5)	0.76 (0.44)	−0.06 (−0.14, 0.01)	0.0966	0.5669	1.06 (0.38)	0.95 (0.42)	0.11 (−0.21, 0.43)	0.4585	0.8158	0.5400
C18 negative	129.0193	21	citraconate	M−H	7.85 (4.78)	6.97 (4.1)	0.88 (0.22, 1.53)	0.0092	0.2936	9.18 (5.15)	7.99 (4.52)	1.19 (0.18, 2.2)	0.0211	0.6034	5.85 (3.3)	5.35 (2.93)	0.5 (−0.26, 1.25)	0.1965	0.6299	7.79 (4.83)	7.76 (2.1)	0.02 (−3.42, 3.46)	0.9888	0.9888	0.5139
C18 negative	167.0342	21.3	homogentisate	M−H	0.0179 (0.0063)	0.0191 (0.0079)	−0.0012 (−0.0022, −2e-04)	0.0176	0.4296	0.0177 (0.005)	0.0174 (0.0044)	3e-04 (−6e-04, 0.0012)	0.5156	0.7920	0.0189 (0.0077)	0.0225 (0.0106)	−0.0036 (−0.0057, −0.0014)	0.0015	0.1853	0.0116 (0.0034)	0.0117 (0.0035)	−1e-04 (−0.0026, 0.0024)	0.9493	0.9794	**0.0052**
HILIC positive	147.0764	77.1	glutamine	M+H	277.58 (104.41)	290.43 (105.81)	−12.85 (−23.76, −1.94)	0.0212	0.4296	286.62 (81.43)	297.71 (92.68)	−11.09 (−22.6, 0.41)	0.0586	0.6034	256.46 (130.74)	267.08 (118.42)	−10.63 (−32.72, 11.47)	0.3424	0.7518	347.94 (66.22)	406.34 (59.54)	−58.4 (−117.57, 0.77)	0.0525	0.7457	0.3018
HILIC positive	114.0662	36.1	creatinine	M+H	37.34 (14.14)	38.71 (13.96)	−1.37 (−2.55, −0.18)	0.0237	0.4296	40.26 (13.75)	41.18 (14.19)	−0.92 (−2.43, 0.58)	0.2280	0.6354	32.37 (13.47)	34.94 (13.07)	−2.57 (−4.56, −0.59)	0.0115	0.4117	42.89 (13.27)	39.21 (11.94)	3.68 (−3.54, 10.9)	0.2825	0.7457	0.1629
HILIC positive	120.0032	65.8	glycine	M+2Na−H	105.15 (41.2)	109.92 (42.22)	−4.76 (−9.27, −0.25)	0.0385	0.5178	103.76 (36.06)	107.09 (38.82)	−3.33 (−9.7, 3.04)	0.3034	0.7136	104.39 (48.71)	110.79 (47.23)	−6.4 (−12.97, 0.18)	0.0563	0.4578	131.97 (20.83)	141.66 (23.99)	−9.69 (−34.74, 15.37)	0.4092	0.8031	0.7512
HILIC positive	269.2257	27.5	retinol	M−H2O+H	6.72 (2.97)	6.39 (2.56)	0.33 (0.01, 0.64)	0.0406	0.5178	7.78 (2.91)	7.33 (2.59)	0.45 (0, 0.9)	0.0511	0.6034	5.19 (2.44)	5.14 (1.94)	0.05 (−0.39, 0.5)	0.8116	0.9539	6.01 (1.75)	4.78 (0.75)	1.22 (−0.23, 2.68)	0.0905	0.7457	0.2101
HILIC positive	163.123	41.4	nicotine	M+H	0.02	0.03 (0.1)	−0.01 (−0.03, 0)	0.0423	0.5178	0.01 (0.03)	0.02 (0.05)	−0.01 (−0.01, 0)	0.2303	0.6354	0.03 (0.1)	0.05 (0.14)	−0.02 (−0.05, 0.01)	0.1588	0.6299	0 (0)	0.06 (0.21)	−0.06 (−0.2, 0.08)	0.3379	0.7599	0.4473

SD: Standard Deviation; CI: Confidence Interval; FDR: False Discovery Rate. Heterogeneity P values were calculated using Cochran’s Q test from a random-effects meta-analysis of the site-specific effect estimates from Brazil, India, and South Africa

**Table 4.** Top Associations between Targeted Metabolites with Confirmed Annotations and TB Resistance in the Country-Specific Cohorts. Quantified Concentrations in uM were Analyzed. Metabolites with P Value < 0.05 or False Discovery Rate < 0.2 in Each Country-Specific Analysis are Shown.

Cohort	Platform	m/z	time	Name	Adduct	Resister Mean (SD)	Control Mean (SD)	Difference Resister vs. Control (95% CI)	P	FDR
Brazil (155 pairs)	HILIC positive	146.1176	45.6	butyrobetaine	M+H	0.77 (0.34)	0.84 (0.4)	−0.07 (−0.12, −0.02)	0.0056	0.5046
	C18 negative	391.2857	149.3	chenodeoxycholic acid/deoxycholic acid	M−H	0.17 (0.2)	0.3 (0.56)	−0.12 (−0.22, −0.03)	0.0117	0.5046
	C18 negative	105.0193	20.2	glyceric acid	M−H	4.4 (2.5)	3.73 (2.43)	0.66 (0.15, 1.18)	0.0119	0.5046
	C18 negative	129.0193	21	2-methylmaleate	M−H	9.18 (5.15)	7.99 (4.52)	1.19 (0.18, 2.2)	0.0211	0.6034
	C18 negative	329.2484	239.2	docosapentaenoic acid	M−H	88.52 (61.98)	74.92 (50.66)	13.6 (1.78, 25.41)	0.0244	0.6034
	C18 negative	227.2015	225	myristic acid	M−H	7.74 (7.15)	6.38 (5.19)	1.36 (0.06, 2.66)	0.0404	0.6034
	C18 negative	303.2328	235.3	arachidonic acid	M−H	1393.92 (915.3)	1210.12 (750.43)	183.8 (7.88, 359.72)	0.0407	0.6034
	HILIC positive	205.0683	56.3	mannitol	M+Na	0.87 (0.73)	1.25 (2.22)	−0.37 (−0.73, −0.01)	0.0415	0.6034
India (103 pairs)	C18 negative	167.0342	21.3	homogentisate	M−H	0.0189 (0.0077)	0.0225 (0.0106)	−0.0036 (−0.0057, −0.0014)	0.0015	**0.1853**
	HILIC positive	114.0662	36.1	creatinine	M+H	32.37 (13.47)	34.94 (13.07)	−2.57 (−4.56, −0.59)	0.0115	0.4117
	HILIC positive	209.0916	53.1	kynurenine	M+H	1.34 (0.36)	1.43 (0.4)	−0.09 (−0.17, −0.02)	0.0170	0.4117
	HILIC positive	344.2792	27.5	lauroylcarnitine	M+H	0.07 (0.05)	0.09 (0.07)	−0.02 (−0.03, 0)	0.0186	0.4117
	HILIC positive	316.2481	28.5	decanoylcarnitine	M+H	0.14 (0.15)	0.18 (0.19)	−0.04 (−0.07, −0.01)	0.0217	0.4117
	C18 negative	167.021	20.5	urate	M−H	133.52 (52.06)	147.36 (63.3)	−13.85 (−25.84, −1.85)	0.0242	0.4117
	C18 negative	181.0508	22.2	hydroxyphenyllactate	M−H	1.32 (0.47)	1.44 (0.52)	−0.12 (−0.22, −0.02)	0.0242	0.4117
	HILIC positive	189.1234	64	acetyllysine	M+H	0.03 (0.05)	0.02 (0.03)	0.01 (0, 0.01)	0.0273	0.4117
	HILIC positive	583.2548	28.7	biliverdin	M+H	5.12 (5.55)	6.66 (6.98)	−1.55 (−2.94, −0.16)	0.0292	0.4117
	HILIC positive	130.0863	58.7	pipecolate	M+H	9.81 (4.96)	11.18 (5.66)	−1.38 (−2.68, −0.07)	0.0394	0.4578
	HILIC positive	260.1854	35.9	hexanoylcarnitine	M+H	0.07 (0.05)	0.08 (0.06)	−0.01 (−0.02, 0)	0.0455	0.4578
	HILIC positive	205.0683	56.3	mannitol	M+Na	1.44 (1.22)	1.19 (0.52)	0.25 (0, 0.49)	0.0472	0.4578
South Africa (11 pairs)	HILIC positive	132.0656	70.7	hydroxyproline	M+H	10.3 (5.64)	16.8 (7.88)	−6.51 (−10.23, −2.78)	0.0030	0.3812
	C18 negative	188.0566	20.5	n-acetylglutamic acid	M−H	0.0023 (0.0011)	0.0033 (0.0012)	−0.001 (−0.0018, −3e-04)	0.0134	0.7457
	C18 negative	243.0621	23.5	uridine	M−H	3.71 (0.59)	3.12 (0.62)	0.59 (0.03, 1.16)	0.0410	0.7457
	HILIC positive	153.0407	44.9	xanthine	M+H	1.66 (0.5)	1.25 (0.47)	0.41 (0, 0.81)	0.0481	0.7457

SD: Standard Deviation; CI: Confidence Interval; FDR: False Discovery Rate.

## Data Availability

Deidentified participant-level data are not publicly available because of participant consent, institutional review board requirements, and data-use restrictions across the participating cohorts. Requests for access may be submitted to the corresponding author and will be considered by the relevant study investigators and institutions, subject to applicable approvals and data-use agreements. Summary-level metabolomics results are provided in the Supplementary Materials.

## References

[1] Pai M, Behr MA, Dowdy D, Tuberculosis. Nat Rev Dis Primers 2016; 2: 16076. 10.1038/nrdp.2016.76 27784885

[2] Baliashvili D, Gandhi NR, Kim S, Resistance to Mycobacterium tuberculosis Infection Among Household Contacts: A Multinational Study. Clin Infect Dis 2021; 73(6): 1037–45. 10.1093/cid/ciab269 PMC8442792 33772550

[3] Verrall AJ, Netea MG, Alisjahbana B, Hill PC, van Crevel R. Early clearance of Mycobacterium tuberculosis: a new frontier in prevention. Immunology 2014; 141(4): 506–13. 10.1111/imm.12223 PMC3956425 24754048

[4] Stein CM, Nsereko M, Malone LL, Long-term Stability of Resistance to Latent Mycobacterium tuberculosis Infection in Highly Exposed Tuberculosis Household Contacts in Kampala, Uganda. Clinical infectious diseases : an official publication of the Infectious Diseases Society of America 2019; 68(10): 1705–12. 10.1093/cid/ciy751 PMC6495009 30165605

[5] Howard NC, Khader SA. Immunometabolism during Mycobacterium tuberculosis Infection. Trends Microbiol 2020; 28(10): 832–50. 10.1016/j.tim.2020.04.010 PMC7494650 32409147

[6] Yu Y, Jiang XX, Li JC. Biomarker discovery for tuberculosis using metabolomics. Front Mol Biosci 2023; 10: 1099654. 10.3389/fmolb.2023.1099654 PMC9986447 36891238

[7] Collins JM, Siddiqa A, Jones DP, Tryptophan catabolism reflects disease activity in human tuberculosis. JCI Insight 2020; 5(10). 10.1172/jci.insight.137131 PMC7259525 32369456

[8] Long NP, Anh NK, Yen NTH, Comprehensive lipid and lipid-related gene investigations of host immune responses to characterize metabolism-centric biomarkers for pulmonary tuberculosis. Sci Rep 2022; 12(1): 13395. 10.1038/s41598-022-17521-4 PMC9352691 35927287

[9] Pretorius C, Luies L. Characterising the urinary acylcarnitine and amino acid profiles of HIV/TB co-infection, using LC-MS metabolomics. Metabolomics 2024; 20(5): 92. 10.1007/s11306-024-02161-8 PMC11297823 39096437

[10] Simmons JD, Van PT, Stein CM, Monocyte metabolic transcriptional programs associate with resistance to tuberculin skin test/interferon-gamma release assay conversion. J Clin Invest 2021; 131(14). 10.1172/JCI140073 PMC8279582 34111032

[11] Tripathi D, Devalraju KP, Neela VSK, Metabolites enhance innate resistance to human Mycobacterium tuberculosis infection. JCI Insight 2022; 7(22). 10.1172/jci.insight.152357 PMC9746823 36509283

[12] Hamilton CD, Swaminathan S, Christopher DJ, RePORT International: Advancing Tuberculosis Biomarker Research Through Global Collaboration. Clin Infect Dis 2015; 61Suppl 3(Suppl 3): S155–9. 10.1093/cid/civ611 PMC4583572 26409277

[13] Gandhi NR, Fernandes Gyorfy M, Paradkar M, Novel Genetic Locus Associated with Resistance to M. tuberculosis Infection: A Multi-Ancestry Genome-Wide Association Study. medRxiv 2026.

[14] Go YM, Walker DI, Liang Y, Reference Standardization for Mass Spectrometry and High-resolution Metabolomics Applications to Exposome Research. Toxicol Sci 2015; 148(2): 531–43. 10.1093/toxsci/kfv198 PMC4675836 26358001

[15] Liu KH, Nellis M, Uppal K, Reference Standardization for Quantification and Harmonization of Large-Scale Metabolomics. Anal Chem 2020; 92(13): 8836–44. 10.1021/acs.analchem.0c00338 PMC7887762 32490663

[16] Liu C, Scorr L, Kilic-Berkmen G, A metabolomic study of cervical dystonia. Parkinsonism Relat Disord 2021; 82: 98–103. 10.1016/j.parkreldis.2020.11.020 PMC7856090 33271463

[17] Johnson JM, Yu T, Strobel FH, Jones DP. A practical approach to detect unique metabolic patterns for personalized medicine. Analyst 2010; 135(11): 2864–70. 10.1039/c0an00333f PMC3069708 20838665

[18] Soltow QA, Strobel FH, Mansfield KG, Wachtman L, Park Y, Jones DP. High-performance metabolic profiling with dual chromatography-Fourier-transform mass spectrometry (DC-FTMS) for study of the exposome. Metabolomics 2013; 9(1 Suppl): S132–S43. 10.1007/s11306-011-0332-1 PMC4517297 26229523

[19] Yu T, Park Y, Johnson JM, Jones DP. apLCMS--adaptive processing of high-resolution LC/MS data. Bioinformatics 2009; 25(15): 1930–6. 10.1093/bioinformatics/btp291 PMC2712336 19414529

[20] Uppal K, Walker DI, Jones DP. xMSannotator: An R Package for Network-Based Annotation of High-Resolution Metabolomics Data. Anal Chem 2017; 89(2): 1063–7. 10.1021/acs.analchem.6b01214 PMC5447360 27977166

[21] Wishart DS, Tzur D, Knox C, HMDB: the Human Metabolome Database. Nucleic Acids Res 2007; 35(Database issue): D521–6. 10.1093/nar/gkl923 PMC1899095 17202168

[22] Schymanski EL, Jeon J, Gulde R, Identifying small molecules via high resolution mass spectrometry: communicating confidence. Environ Sci Technol 2014; 48(4): 2097–8. 10.1021/es5002105 24476540

[23] Benjamini Y, Hochberg Y. Controlling the False Discovery Rate: A Practical and Powerful Approach to Multiple Testing. Journal of the Royal Statistical Society Series B (Methodological) 1995; 57(1): 289–300.

[24] Mehta A, Liu C, Nayak A, Untargeted high-resolution plasma metabolomic profiling predicts outcomes in patients with coronary artery disease. PLoS One 2020; 15(8): e0237579. 10.1371/journal.pone.0237579 PMC7444579 32810196

[25] Islam SJ, Liu C, Mohandas AN, Metabolomic signatures of ideal cardiovascular health in black adults. Sci Rep 2024; 14(1): 1794. 10.1038/s41598-024-51920-z PMC10799852 38245568

[26] Li S, Park Y, Duraisingham S, Predicting network activity from high throughput metabolomics. PLoS Comput Biol 2013; 9(7): e1003123. 10.1371/journal.pcbi.1003123 PMC3701697 23861661

[27] Phuoc Long N, Heo DY, Park S, Molecular perturbations in pulmonary tuberculosis patients identified by pathway-level analysis of plasma metabolic features. PLoS One 2022; 17(1): e0262545. 10.1371/journal.pone.0262545 PMC8786114 35073339

[28] Kang PB, Azad AK, Torrelles JB, The human macrophage mannose receptor directs Mycobacterium tuberculosis lipoarabinomannan-mediated phagosome biogenesis. J Exp Med 2005; 202(7): 987–99. 10.1084/jem.20051239 PMC2213176 16203868

[29] Rajaram MVS, Arnett E, Azad AK, M. tuberculosis-Initiated Human Mannose Receptor Signaling Regulates Macrophage Recognition and Vesicle Trafficking by FcRgamma-Chain, Grb2, and SHP-1. Cell Rep 2017; 21(1): 126–40. 10.1016/j.celrep.2017.09.034 PMC5960073 28978467

[30] Fiorucci S, Biagioli M, Zampella A, Distrutti E. Bile Acids Activated Receptors Regulate Innate Immunity. Front Immunol 2018; 9: 1853. 10.3389/fimmu.2018.01853 PMC6099188 30150987

[31] Chen ML, Takeda K, Sundrud MS. Emerging roles of bile acids in mucosal immunity and inflammation. Mucosal Immunol 2019; 12(4): 851–61. 10.1038/s41385-019-0162-4 30952999

[32] Lee W, VanderVen BC, Fahey RJ, Russell DG. Intracellular Mycobacterium tuberculosis exploits host-derived fatty acids to limit metabolic stress. J Biol Chem 2013; 288(10): 6788–800. 10.1074/jbc.M112.445056 PMC3591590 23306194

[33] Pandey AK, Sassetti CM. Mycobacterial persistence requires the utilization of host cholesterol. Proc Natl Acad Sci U S A 2008; 105(11): 4376–80. 10.1073/pnas.0711159105 PMC2393810 18334639

[34] Daniel J, Maamar H, Deb C, Sirakova TD, Kolattukudy PE. Mycobacterium tuberculosis uses host triacylglycerol to accumulate lipid droplets and acquires a dormancy-like phenotype in lipid-loaded macrophages. PLoS Pathog 2011; 7(6): e1002093. 10.1371/journal.ppat.1002093 PMC3121879 21731490

[35] Chen M, Divangahi M, Gan H, Lipid mediators in innate immunity against tuberculosis: opposing roles of PGE2 and LXA4 in the induction of macrophage death. J Exp Med 2008; 205(12): 2791–801. 10.1084/jem.20080767 PMC2585850 18955568

[36] Collins JM, Wassie L, Bobosha K, Infection with Mycobacterium tuberculosis depletes host fatty acids in humans and non-human primates. eBioMedicine 2026; 125: 106193. 10.1016/j.ebiom.2026.106193 PMC12964237 41763182

[37] Bagnato G, Iulianelli A, Sanna A, Basile A. Glycerol Production and Transformation: A Critical Review with Particular Emphasis on Glycerol Reforming Reaction for Producing Hydrogen in Conventional and Membrane Reactors. Membranes (Basel) 2017; 7(2). 10.3390/membranes7020017 PMC5489851 28333121

[38] TeSlaa T, Bartman CR, Jankowski CSR, The Source of Glycolytic Intermediates in Mammalian Tissues. Cell Metab 2021; 33(2): 367–78 e5. 10.1016/j.cmet.2020.12.020 PMC8088818 33472024

[39] Singh D, Rai V, Agrawal DK. Regulation of Collagen I and Collagen III in Tissue Injury and Regeneration. Cardiol Cardiovasc Med 2023; 7(1): 5–16. 10.26502/fccm.92920302 PMC9912297 36776717

[40] Zhai Y, Ren J, Ding Z, The diagnostic value of hydroxyproline combined with tuberculosis infection T lymphocyte spot assay in pulmonary tuberculosis. J Thorac Dis 2024; 16(10): 7052–62. 10.21037/jtd-24-1585 PMC11565317 39552900

[41] Leung IK, Krojer TJ, Kochan GT, Structural and mechanistic studies on gamma-butyrobetaine hydroxylase. Chem Biol 2010; 17(12): 1316–24. 10.1016/j.chembiol.2010.09.016 21168767

[42] Koeth RA, Levison BS, Culley MK, gamma-Butyrobetaine is a proatherogenic intermediate in gut microbial metabolism of L-carnitine to TMAO. Cell Metab 2014; 20(5): 799–812. 10.1016/j.cmet.2014.10.006 PMC4255476 25440057

[43] Nieto-Caballero VE, Reijneveld JF, Ruvalcaba A, History of tuberculosis disease is associated with genetic regulatory variation in Peruvians. PLoS Genet 2024; 20(6): e1011313. 10.1371/journal.pgen.1011313 PMC11208071 38870230

